# Perceiving Social-Emotional Volatility and Triggered Causes of COVID-19

**DOI:** 10.3390/ijerph18094591

**Published:** 2021-04-26

**Authors:** Si Jiang, Hongwei Zhang, Jiayin Qi, Binxing Fang, Tingliang Xu

**Affiliations:** 1 Key Laboratory of Trustworthy Distributed Computing and Service (BUPT), Ministry of Education, Beijing 100876, China; jiangsi@bupt.edu.cn (S.J.); fangbx@bupt.edu.cn (B.F.); 2 School of Cyberspace Security, Beijing University of Posts and Telecommunications, Beijing 100876, China; 3 Center for Intelligence Science and Technology, Beijing University of Posts and Telecommunications, Beijing 100876, China; zhwzss@163.com; 4 Institute of Artificial Intelligence and Change Management, Shanghai University of International Business and Economics, Shanghai 200336, China; 5 Cyberspace Institute of Advanced Technology, Guangzhou University, Guangzhou 510006, China; 6 College of Agriculture and Animal Husbandry, Qinghai University, Xining 810016, China; 2020990037@qhu.edu.cn

**Keywords:** social-emotional volatility, COVID-19, cause detection, deep learning

## Abstract

Health support has been sought by the public from online social media after the outbreak of novel coronavirus disease 2019 (COVID-19). In addition to the physical symptoms caused by the virus, there are adverse impacts on psychological responses. Therefore, precisely capturing the public emotions becomes crucial to providing adequate support. By constructing a domain-specific COVID-19 public health emergency discrete emotion lexicon, we utilized one million COVID-19 theme texts from the Chinese online social platform Weibo to analyze social-emotional volatility. Based on computed emotional valence, we proposed a public emotional perception model that achieves: (1) targeting of public emotion abrupt time points using an LSTM-based attention encoder-decoder (LAED) mechanism for emotional time-series, and (2) backtracking of specific triggered causes of abnormal volatility in a cognitive emotional arousal path. Experimental results prove that our model provides a solid research basis for enhancing social-emotional security outcomes.

## 1. Introduction

The pandemic of the novel coronavirus disease 2019 (COVID-19) highlights the vital role of online social media in the development of sudden public health. When the public is in home isolation (to avoid outdoor gathering activities and to reduce chances of infection), people are more likely to share on social networks benign symptoms and conditions (e.g., headache, stress, and cough), as well as inconveniences associated with those conditions [1]. These psychological pressures affect people’s physical and mental health and pose a potential threat to normal and stable society. 

Several scholars have been concerned about the psychological impact and have identified that the media played a vital role during the COVID-19 pandemic because the public sought security through excessive use of Internet information related to the events [2,3,4]. To supplement previous research on psychological effects studying the polarity of emotions [5], which are normally considered to be positive, negative, or neutral [6], discrete emotions have predictable effects on subsequent cognitive effort, depending on an individual’s underlying appraisal of certainty [7]. Thus, we utilized eight discrete emotional dimensions to measure changes in the public’s psychological state on Weibo, a Chinese version of Twitter and a platform that appeals to the 500 million people sharing their opinions. Consequently, we were able to dynamically perceive the emotional volatility of a discrete emotional time-series of collective cognition since social network data are the most effective and accurate indicators for studying public sentiment [8]. 

**Figure 1:**
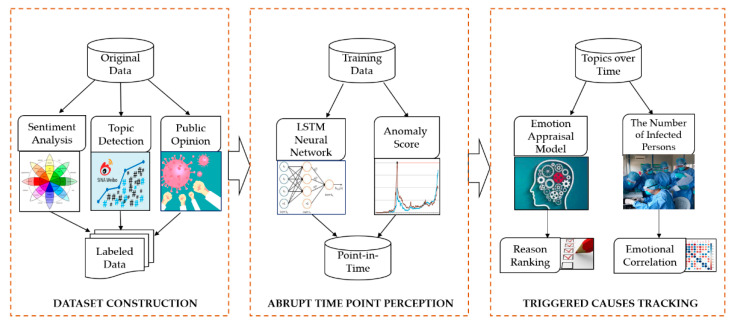
Overall Public Emotional Perception Model.

We integrated both abnormal emotional volatility detection and triggered causes into a perception model to dynamically monitor the public’s emotional reactions. The volatility is reflected in emotional valence, which is generally considered to be an expression of emotional intensity and strength. We first constructed a discrete emotion lexicon that for measured and monitored the public’s emotional state with regard to COVID-19 public health emergencies. In addition, we proposed a public emotional perception model that integrated deep learning to detect abrupt timing by the stimulus. Figure 1 shows the overall model process. First, we constructed a series of datasets, including the computed emotional valences, topics in the pandemic period, and tags for events that aroused a high degree of public attention. These labeled data served as the basis for the model for evaluating emotion. Second, we input each continuous emotional time-series and their valences into our proposed LSTM-based attention encoder-decoder (LAED) mechanism on emotional burst time points estimation. Third, we traced back the time window before that time point, followed the emotional arousal path forward according to the emotion appraisal model, ranked the specific corresponding causes of emotional volatility, and finally accurately positioned the largest risks affecting public emotions. We also compared the Pearson correlation coefficient between infected persons and discrete emotions. 

Experimental results show that our domain-specific emotional lexicon can capture the public emotional situation, and the perception model not only provides a solution to real-time dynamic monitoring of the public’s emotional state but also serves as an aid for detecting actual emotional causes. Public health emergencies concern the safety of people’s livelihood, and our research provides a solution using the online social media context to identify in advance potential emotional risks. 

The remainder of this paper is organized as follows: Section 2 summarizes related research work, Section 3 describes the methods of domain-specific discrete emotion lexicon construction and our proposed time-point detection and cause perception model, Section 4 reports the experimental process and results, and the conclusions are presented in Section 5.

## 2. Related Work

In contrast to the existing studies on event evolution and emotion classification, we managed to solve how specific discrete emotions depict the psychological level of the public during an emergency. We refined the relevant research in the two subsequent sections: Section 2.1 public emotion and discrete emotion lexicon and Section 2.2 emotional abrupt and cause detection.

### 2.1. Public Emotion and Discrete Emotion Lexicon

Lexicon-based sentiment analysis has shown promise for analyzing the impact of user emotions on social networks [9,10,11]. Therefore, it is imperative to build an adaptive emotional lexicon to capture the public’s discrete emotions. TensiStrength detects stress and relaxation strength in social web texts associated with human-annotated corpora [12]. Using the correlation of online users’ stress states from Twitter interactions, a hybrid model comprising a factor graph model and a convolutional neural network (CNN) was proposed to improve detection performance [13]. For new words in the field, a large unlabeled corpus by Sogou, a Chinese search engine, was utilized to correct and expand current Chinese sentiment dictionaries [14], and a deep learning neural network extracted keywords to predict and identify potential default risk platforms [15]. However, none of these studies were conducted on specific Chinese discrete emotions. The most relevant Chinese fine-grained scheme initially proposed by Ren-CECps [16] ensures the accuracy of emotive words but lacks the ability to adapt to the domain. They constructed a new lexicon, Ren-CECps-SWB 2.0, for measuring the subjective well-being (SWB) of Chinese [17]. 

To this end, it was necessary to construct an emotional lexicon based on human cognitive psychology, which is suitable for accurately describing the changes of public emotional and stress states in online social media during the COVID-19 pandemic.

### 2.2. Emotional Abrupt and Cause Detection

There is a lack of research on the specific factors influencing public mentality during the COVID-19 pandemic [3], and the research has failed to solve the problem of tracking and identifying the stressors to explain the role of anxiety from the perspective of psychological factors [18]. Traditional cause extraction involves finding the event corresponding to a particular emotion by using the grammatical rules of emotional sentences, using a multi-attention-based neural network that was grounded in the context of words to locate emotional clauses [19]. 

However, our definition of the cause of emotion is different from the prior ones. We maintain that emotion at the semantic level is the description of the emotional body and emotional target object, which can be achieved by syntactic and lexical analysis. The emotional cause was proposed based on the OCC model of emotion [20], a rule constructed by Bayesian probability to recognize different emotional components under different emotional factors [21]. Some researchers analyzed emotional changes, extracted foreground and background latent dirichlet allocation (LDA) models to extract foreground topics, and proposed cause candidate and background LDA models to rank the popularity of extracted foreground topics in the emotional change period [22]. Others adopted a multivariate Gaussian distribution with a power-law distribution to model and analyze the users’ emotion of microblogs and to detect the reactive emotion state [23], focusing on the sentiment tracking of the needs of different entities and detecting sentiment spikes on the problem of extracting and ranking the causes of a sentiment spike [24].

Therefore, we detected the most obvious type of emotions among the public due to their different psychological acceptance of media information and government measures, analyzed the effect of different causes of abnormal emotion, and emphasized the most extensive public risk factors.

## 3. Methods

### 3.1. Dataset Analysis

The experiment selected the 230 COVID-19 themes dataset (https://www.datafountain.cn/competitions/423, accessed on 3 March 2020) for the period between 1 January 2020 and 20 February 2020, during which time a total of 1 million Chinese Weibo data were collected. We calculated the emotional categories and emotional valences of different individual public posts within the unit time interval to integrate the public collective emotions and detect hot events. To cover all of the candidate events, we referred to an emerging social media site, zhiweidata.com, as well as the traditional media Nanfang Metropolis Daily “Special COVID-19”. We performed the LDA topic model in the test corpus to make the detection of epidemic events more representative. For the evaluation of perplexity and coherence score, the optimal number of topics equaled 7. Other relevant reports had different divisions on the number and type of topics according to the contents and objects. Our research team individually labeled the major COVID-19 events into topics by personal cognition of real events, in addition to topic classifications made by news reports. Finally, we summarized 7 topics (listed in Table 2) based on the content from the open data we used and used the LDA topic model to label the corpus by the dominant percentage score. Meanwhile, the time points were labeled in the form of text similarity to major events and served as the basis for our response to the public. 

### 3.2. Measurement of Public Emotions

Regarding the COVID-19 public health emergency, a suitable emotion lexicon was the basis for measuring the public’s emotional characteristics. Ren_CECps lexicon consists of four positive emotions—expect, joy, love, surprise—and four negative emotions—anxiety, sorrow, angry, hate—providing a Chinese annotation model with eight emotional dimensions. We extracted labeled keywords and emotional phrases from the original Ren_CECps lexicon and loaded them on a 10,000 randomly extracted test corpus. The results indicated that the coverage was only 14.85% of the corpus, which suggests that the original lexicon lacked the capability of understanding the actual public emergency emotional situation. To better adapt and measure public opinion of the COVID-19 outbreak, we adapted a domain discrete emotion lexicon, optimized emotional volatility, and finally constructed a better lexicon that could more accurately describe the public emotional phenomenon.

#### 3.2.1. Discrete Emotion Lexicon Construction 

We extracted labeled keywords and phrases with eight-dimensional emotion tags from 1487 Chinese blogs in Ren-CECps, checked word length, part of speech, and emotional intensity and eliminated repeated words that had no clear meaning when the eight-dimensional emotional scores were all equal to 0. Then, extending synonyms from HIT Tongyici Cilin were extended by bootstrapping to add synonyms to the list of seeds, and the list was continuously updated. To adjust for repeated words, we adopted the method of multiple sampling, which is updated in Equation (1):

(1) $$e_{i}=\frac{1}{N}\overset{M}{\underset{j=1}{\sum }}e_{Valence}^{j}$$

where, $e_{i}$ represents valence of the emotional type, N is the total number under the emotional type, $e_{Valence}$ is the original annotated emotional strength, and M is the total number of words appearing. Previous experimental experience proves that our approach of computing emotional valence for eight emotional dimensions within the time interval can express emotion volatility [17,25]. 

Secondly, we combined context representation by using the Google open source tool Word2vec and the CBOW language model. We trained the semantic word vectors from 3.5G SogouCA (http://www.sogou.com/labs/resource/ca.php, accessed on 20 June 2017) and satisfied the maximum conditional probability. In addition, we extracted high-frequency word sequences of the test corpus and expanded the domain emotion lexicon by word vector similarity. On the basis of calculating the cosine distance, we used the *k*-NN algorithm to locate the most relevant candidate words that matched the target words. By adjusting the threshold range of *k*, we determined the emotional category of the new candidate words and assigned value to them. After repeated experiments and manual verification, we chose *k* = 5 given the premise of similarity greater than 0.6, and finally expanded to 2455 domain-specific extra words on the topic of the COVID-19 public health pandemic.

**Figure 2:**
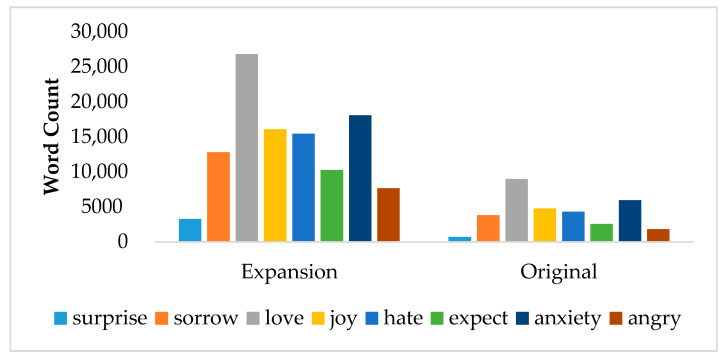
Comparison of the Lexicon Scale of Expansion and the Original.

After the above steps, we constructed a total of 45,096 emotion vectors with 110,387 overlapping dimensions. Figure 2 shows the lexicon scale comparison of expansion and origin in the number of word count in each emotion. The scale of the expanded emotional lexicon was nearly 4-fold of the original. 

#### 3.2.2. Optimization Strategy of Social-Emotional Volatility

To verify the expanded lexicon validity, we extracted an extra 10,000 test corpus from the open dataset. First, we cleaned low-frequency words (word frequency less than 5) from the lexicon. Following the experience of previous work, volatility was considered with different levels of valence. With the increase in valence, the number of words in the lexicon and domain words decreased continuously, but the effect of emotional volatility was more noticeable under the same situation. Because a value of 0 in a dimension means there is no emotional expression in the corresponding dimension, we removed the non-zero valence lower than a certain threshold for all eight dimensions. Then, we statistically compared the original with the expansion, as well as with the words retrieved in the emotion lexicon, the coverage of the test corpus, and the count and coverage of COVID-19 domain words under different valence thresholds in Table 1.

The experiment showed that the expanded domain lexicon significantly improved the matching of the COVID-19 corpus compared with the original. The coverage of both the domain words and the test corpus increased 5-fold, reaching 74.84%. After testing and observing the change in emotional volatility under each threshold, the coverage rate of the corpus and field decreased significantly after 0.4. Therefore, we chose 0.3 as our optimized threshold, where the coverage rate of the corpus was still close to 50% under the condition of 42.52% coverage rate of field words, and the emotional volatility of eight dimensions was still relatively obvious.

We observed whether the improved lexicon was consistent with the emotional fluctuations of the public. For each microblog content, there is also the phenomenon of emotional co-occurrence. This causes the valence in each dimension to weaken, and it is the reason why we optimized emotional valence to 0.3 to intensify the emotional volatility. The calculation method of emotional valence within a unit time interval E is formulated in Equation (2):

(2) $$E=\frac{1}{M}\sum_{m=1}^{M}\left[\frac{1}{N}\sum_{n=1}^{N}e_{n}^{m}\left(j\right)\right]=\frac{1}{MN}\sum_{m=1}^{M}\sum_{n=1}^{N}e_{n}^{m}\left(j\right)$$

where $e_{n}^{m}\left(j\right)$ represents the emotional valence of the $j^{th}$ valence of the $n^{th}$ word in a total of m texts per unit of time, so $\sum_{m=1}^{M}\sum_{n=1}^{N}e_{n}^{m}\left(j\right)$ denotes the emotional valence of all words in m texts in the $j^{th}$ dimension in a unit of time.

**Figure 3:**
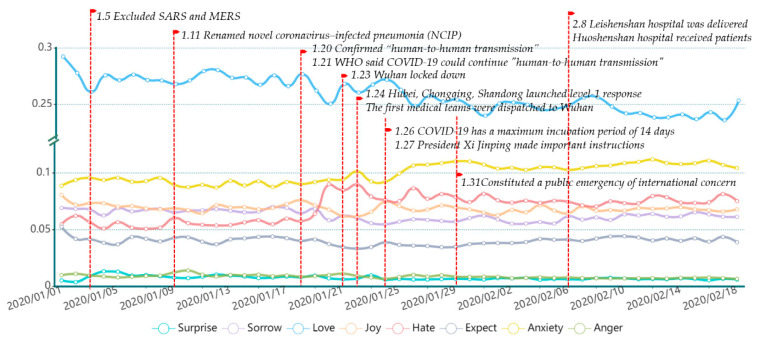
Summary of Eight Emotional Volatilities and Major Events.

We outline the data timeline in Figure 3 to show eight emotional volatilities and their distribution, from 1 January to 18 February. We selected a part of major public opinion events to observe the emotional volatility that reflected the public emotional reactions. We identified the time point, as shown in Figure 3, where the emotion volatility was obvious at the moment of major events. Thus, volatility can accurately depict the emotional state of the public during the pandemic. For example, on 20 January, a news press conference was received with the news of novel coronavirus “Human-to-human transmission”. Afterward, feelings of hate and anxiety dramatically increased and reached an all-time high on 21 January. Overall, negative emotions experienced a significant process of rising emotional valence throughout the entire time period; the most prevalent were hate and anxiety. Love, which had the largest coverage in the lexicon and a high valence, showed an overall downward trend. 

The results show that the adapted emotional lexicon is scientific and effective in psychological quantification. Therefore, as far as discrete emotion research is concerned, it is necessary to analyze the volatility of each emotion carefully to find the types of emotions that are specifically relevant to the events.

### 3.3. Public Emotional Perception Model

#### 3.3.1. Abrupt Time Point Perception

**Figure 4:**
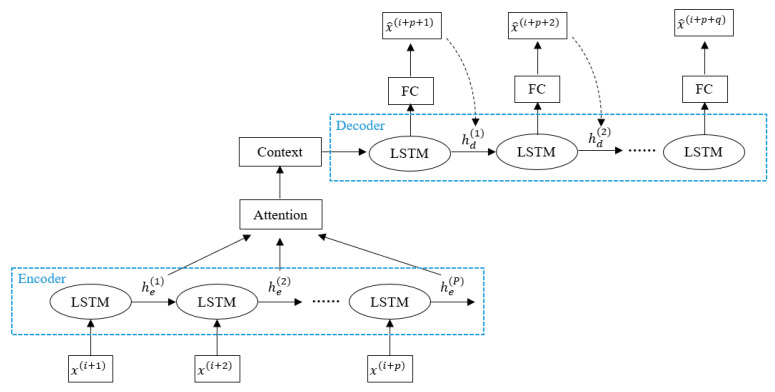
Architecture of LAED Model.

In the midst of a public health security emergency, the public’s emotional state is more sensitive and fluctuates at the time point where the reaction occurs after being stimulated. It is difficult to locate the abrupt time points of public emotions because the data are sparse. This poses a great challenge when trying to create an effective model. Thus, we propose an LSTM-based attention encoder-decoder (LAED) mechanism for emotional time-series to solve this problem, as illustrated in Figure 4.

The model is trained to reconstruct “normal” emotional time-series by taking the most recent emotional time-series instance as the encoder input and jointly predicting several future emotional time-series instances as the decoder output. Then, the reconstruction error between the real target and the decoder output is obtained and used to compute the likelihood of an anomaly at this time point. In the training process, the model only considers a normal instance and predicts a normal instance. The intuition is that when the model encounters new data, including normal and abnormal instances, the reconstruction error of the abnormal emotional valences would be higher than that of the normal ones.

We built a continuous time-series on $H=\left\{x^{\left(1\right)},\;x^{\left(2\right)},\ldots ,x^{\left(D\right)}\right\}$ using January data. Every $x^{\left(i\right)}$ denotes one hour at a time, computed from Section 3.3, where each sequence $x^{\left(i\right)}$ consisted of eight-dimensional emotion vectors at a time instance. Here, we obtained multiple time-series $X_{i}^{p}=\left\{x^{\left(i+1\right)},x^{\left(i+2\right)},\ldots ,x^{\left(i+p\right)}\right\}\left(p\ll D\right)$ by taking a window of length p over a larger time-series H. We first trained the LAED model with a training set that contained no anomalies to reconstruct instances of a normal emotional time-series. The model took the most recent p values as inputs and jointly reconstructed the output future q values ${\hat{X}}_{i+p}^{q}=\left\{{\hat{x}}^{\left(i+p+1\right)},{\hat{x}}^{\left(i+p+2\right)},\ldots ,{\hat{x}}^{\left(i+p+q\right)}\right\}$ with the target emotion time-series $X_{i+p}^{q}=\left\{x^{\left(i+p+1\right)},x^{\left(i+p+2\right)},\ldots ,x^{\left(i+p+q\right)}\right\}$. The model only reconstructed ${\hat{x}}^{\left(i+p+1\right)}$ (i.e., *q* = 1) if the prediction accuracy was low. In this process, $h_{e}^{\left(m\right)}$ is the hidden state of the encoder at step time m for each m∈{1, 2, …, p}, and we utilized the attention mechanism $h_{e}^{\left(m\right)}$ to obtain a context vector representing the sum of different weights on hidden states and to improve prediction accuracy, so the context vector C is represented as follows in Equations (3)–(5):

(3) $$C=\sum_{m=1}^{p}\alpha_{i}h_{e}^{\left(m\right)}$$

(4) $$\alpha =Softmax\left(h_{e}W+b\right)$$

(5) $$h_{e}=\left[h_{e}^{\left(1\right)};\;h_{e}^{\left(2\right)};\ldots ;h_{e}^{\left(p\right)}\right],\;\mathrm{where}\;[;]\;\mathrm{represents}\;\mathrm{concatation}.$$

The context vector C was used for the initial state in the decoder. A fully connected layer on top of the LSTM decoder layer was used to predict the target emotion time-series. During the training process, the decoder used $x^{\left(i+p+n\right)}$ as input to obtain the hidden state $h_{d}^{\left(n+1\right)},\;n\in \left\{1,\;2,\;\ldots ,\;q-1\right\}$ and predicted ${\hat{x}}^{\left(i+p+n+1\right)}$ corresponding to the target $x^{\left(i+p+n+1\right)}$. However, during inference, the predicted value ${\hat{x}}^{\left(i+p+n\right)}$ was input to the decoder to obtain $h_{d}^{\left(n+1\right)}$ to predict ${\hat{x}}^{\left(i+p+n+1\right)}.$ Our model was trained by mean squared error (MSE) as the loss function to measure the distance between $x^{\left(i+p+n+1\right)}$ and ${\hat{x}}^{\left(i+p+n+1\right)}$.

Similar to Malhotra [26], we divided the emotional time-series into four parts: two for normal emotions, N and $V_{N}$, and the other two for abnormal emotions, $V_{A}$ and T. Here, N refers to the training set for the LSTM encoder-decoder model, and $V_{N}$ is used for early stopping to prevent the model from overfitting the training set on N. The reconstruction error vector for step n in the decoder is given by Equation (6):

(6) $$e^{\left(n\right)}=\left|x^{\left(i+p+n\right)}-{\hat{x}}^{\left(i+p+n\right)}\right|$$

Then, we estimated the parameters μ and Σ of a Gaussian distribution N(μ, Σ) by maximum likelihood estimation (MLE) from eight-dimensional reconstruction errors in the normal set $V_{N}$, and set a log probability densities (PD) of errors on $V_{A}$ as anomaly scores, where the lower value indicates a greater likelihood of anomalous emotions. For any $x^{\left(i+p+n\right)}$, the anomaly score is calculated in Equation. (7):

(7) $$S_{n}={\left(e^{\left(n\right)}-\mu \right)}^{T}\Sigma^{-1}\left(e^{\left(n\right)}-\mu \right)$$

where $S_{n}$ is a manual threshold on log PD to separate anomalies from normal data, preventing the possibility of false positives from the test set T. Finally, a separate test set T was used to evaluate the model. 

To separate the abrupt emotional time points from emotional time-series, we utilized a simple and effective threshold adjustment method to find the optimal log PD. Then, we used these log PD against all eight emotional sequences to ensure all the emotions were at the same level for detecting abrupt points. The performance of different log PD on $V_{A}$ in detecting the number of abrupt points is illustrated in Section 4.1.

#### 3.3.2. Triggered Causes Tracking

The purpose of our study was to identify major events that caused an emotion outburst. Because of the limited information available while quarantined at home, the public paid significant attention to the pandemic news. However, depending on the release time of media reports and the transfer behaviors of online users, emotional volatility inevitably delayed or persisted. For this reason, we pinpointed the cause of the sudden change in the preceding period of the time window. Based on what we found, the most obvious emotion types inspired the public in Section 3.3.1, we verified the tracking results from the perspective of semantic features in the context and the perspective of emotion theory, respectively.

From the perspective of semantic features, each event has a unique corresponding eight-dimensional emotional valence; thus, we compared the difference in emotional distribution of the event and of the day. The distribution distance was measured by the Kullback–Leibler divergence [27]. Then, we located the cause ranking, which comes first in the order.

**Figure 5:**
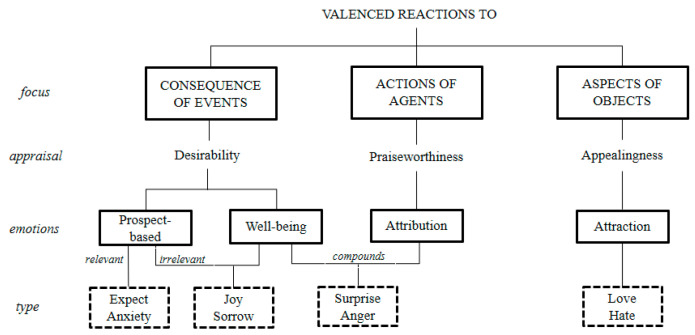
Eight Discrete Emotions Corresponding to the OCC Model.

Emotion theory provides a psychological foundation for human cognition so that the exact discrete emotion is ruled in its elicitation condition. In particular, the OCC theory contains detailed discrete emotions in a context concerning the different consequences of events, actions of agents, and aspects of objects, and OCC theory is widely used as a computational model to depict the human cognition process. It identifies the underlying cognitive structure and emotional dimensions [28]. This approach argues that emotional reactions to an event are a product of personal interpretations and appraisals in a situational environment [29,30]. Therefore, we aligned and divided the eight discrete emotions in our lexicon with the emotional types in the hierarchical structure OCC model, as shown in Figure 5. In the emotional arousal path, we defined the eight emotional types as the emotional pathways of three different concerns in the OCC model, which reflected the three-appraisal event-to-emotion mapping processes and emotional responses. The eight-dimensional emotional response to the *valenced reactions* in the OCC model provided an important theoretical basis for tracing abnormalities. 

Each discrete emotion is elicited by a unique pattern of cognitive appraisals, and situations with the same appraisal pattern will induce the same emotion [31]. At the same time, we marked the candidate events into seven topics and their *focus* in the OCC model in Table 2. 

We computed the emotional distribution of public opinion events and topics according to the abnormal emotions found by the LAED model. We tracked the emotion arousal path to ensure each topic was related to the emotional elicitation conditions. Thus, we obtained the emotional distribution of the candidate scenario in $D_{C}=d_{1}^{C},d_{2}^{C},\ldots ,d_{8}^{C},\;C\in \left\{event,topic,\;day\right\}$. The influence of the corresponding emotional situation of the event on the overall distribution is calculated by KL divergence in Equations (8)–(9):

(8) $$\mathrm{KL}\left(D_{event},D_{day}\right)=\underset{i}{\sum }D_{event}\left(i\right)\log\frac{D_{event}\left(i\right)}{D_{day}\left(i\right)}$$

(9) $$\mathrm{KL}\left(D_{topic},D_{day}\right)=\underset{i}{\sum }D_{topic}\left(i\right)\log\frac{D_{topic}\left(i\right)}{D_{day}\left(i\right)}$$

The method of emotion arousal path highlights the properties of emotion, which identify the issue of finding the most influential event according to the content. Because events are diverse, the emotion arousal path has a fixed induced path from the cognition process, so it more accurately positions the induced causes. The larger the KL value, the greater the difference between the two distributions. In brief, while the situational distribution was the most similar to the overall distribution of the day, we obtained the smallest KL value, which meant that this situation was more likely to influence the public emotion. Thus, we clearly identified emotional spikes when events occurred using the emotional arousal path and target-triggered causes.

For the triggered reasons, we chose the order of emotion-induced ranking metrics in precision in Equation (10) and mean average accuracy (MAP) in Equation (11):

(10) $$Precison@K=\frac{Y_{relevant}}{K}$$

where K refers to the top K texts of the real and predicted ranking, and the number of matched texts in prediction is $Y_{relevant}$. Therefore, assuming that the order of the real matched texts in the prediction $K_{1},K_{2},\ldots ,K_{r}$, where r is the number of all matched texts in the entire list, then:

(11) $$MAP=\frac{\sum_{i=1}^{r}P@K}{\left|r\right|}$$

## 4. Experiment Results and Analyses

In this section, we provide the results of this study. Our emotional perception model was able to position an abrupt time point during COVID-19 that reflected the superiority of our model. Additionally, we include a visual display of pertinent research and reach important conclusions. 

### 4.1. Detection Effect of Abrupt Time Points

The evaluation criteria for the model were geared toward the emotional change detection task. We used the LAED model to detect abnormal emotional valence. Depending on the training of regular “normal” emotions on N, we used the validation set $V_{A}$ to verify the feasibility of the model. Table 3 below shows the average result of different log PD on $V_{A}$ to detect the number of abrupt points in eight emotional time-series.

The set $V_{A}$ contained 1000 emotional time-series with 16 abnormal points. From the table above, the number of predictive abrupt time points decreased as log PD declined, and the true value of abnormal also reduced. The aim was for the model to detect the real abnormal points as much as possible. Although all the abnormal points were found when log PD was set at −3.0, the recall (4/16) was quite low to fit the real points. Therefore, we balanced the accuracy and recall and verified the optimal threshold when log PD was equal to −2.0, where the accuracy of model prediction was 76.9% (10/13) and the recall was 62.5% (10/16). 

**Figure 6:**
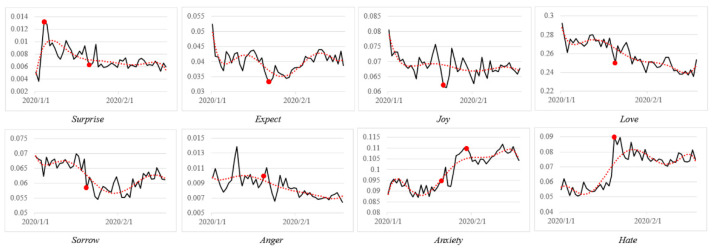
Eight Emotional Volatilities in January and February.

Before we present the LAED model performance, we demonstrate eight emotional volatilities in terms of the day. As shown in in Figure 6, to observe daily emotional volatility, we used the black line to represent the volatility curve of the original emotional valence, and the red dotted line is the polynomial trend line. 

On the top of the figure, all four volatilities are positive emotions, and the bottom are negative emotions. Overall, most emotional valences showed a downward trend in the early two months, as shown by the red dotted line, except for anxiety and hate. There were two main findings:

(1) There was an upward trend of the negative emotions: hate and anxiety. Hate maintained a higher valence from 20 January, when academician Nanshan Zhong announced the phenomenon of “human-to-human” transmission, where we pinpoint a red dot in the figure. This news also triggered anxiety to an obvious increase, with the greatest anxiety identified on 29 January, when all 31 provinces in mainland China launched a first-level response to public health emergencies; thus, we marked them as well. 

Notably, these two events belong to the topic of *pandemic news and data reports* and *scientific breakthroughs and knowledge dissemination*, which correspond to the emotion arousal path of *Aspects of Objects* and *Consequence of Events.* Thus, we believe that hate and anxiety, which differ from other emotions in volatility trends, are more sensitive and accurate in emotional cause tracing.

(2) Almost all positive emotions showed a significant downward trend, where the red dotted lines appear in a similar direction in January. It was on 20 January that all positive emotions dropped sharply in the black volatility line, and almost all of them reached the lowest valence as a result. The difference is that the spike of surprise was quite early on 4 January, when Wuhan reported a total of 44 unexplained cases of viral pneumonia. In the *emotion arousal path,* surprise was identified as a compound appraisal both of *Actions of Agents* and *Consequence of Events*; thus, tied to the reaction of agents and events, where Wuhan was the agent for the public in *focus*. Furthermore, we also found that the trends of sorrow and anger were similar to those of the positives, possibly because the national pandemic prevention measures eased the emotion of the public.

We need to determine the reasons for these abrupt emotional changes based on the LAED model and cognitive emotion arousal path. Although we observed valence according to the results of daily statistics, the abnormal time point cannot be specifically identified owing to the lack of data on a daily basis. We present the model effect of emotional abrupt time point detection based on hourly intervals in months, and use the January data, a total of 744 h, as an example. The reason we selected January data was because COVID-19 was unknown during the initial stage. It provided a comparison of emotional volatility before and after policy control and social governance.

**Figure 7:**
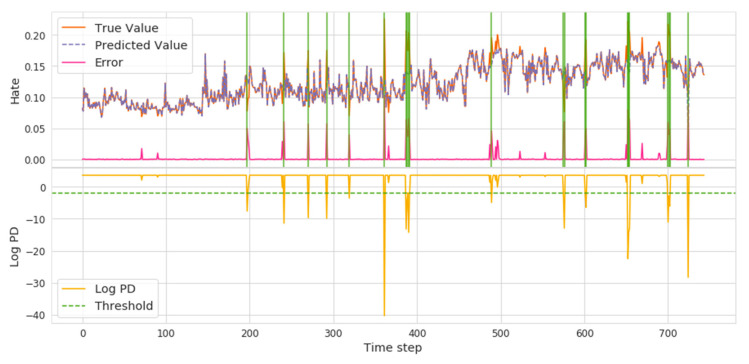
Hate Volatility and Abrupt Time Points.

**Figure 8:**
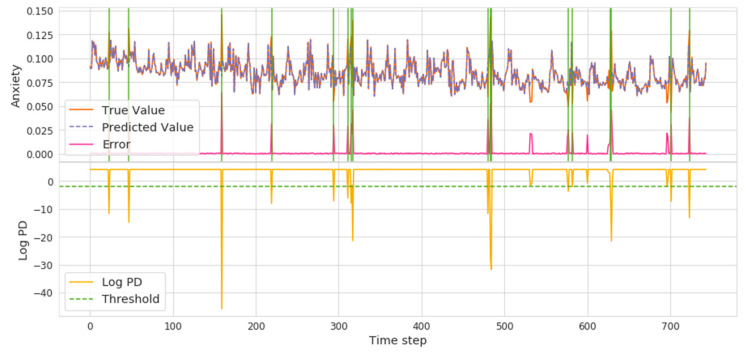
Anxiety Volatility and Abrupt Time Points.

**Figure 9:**
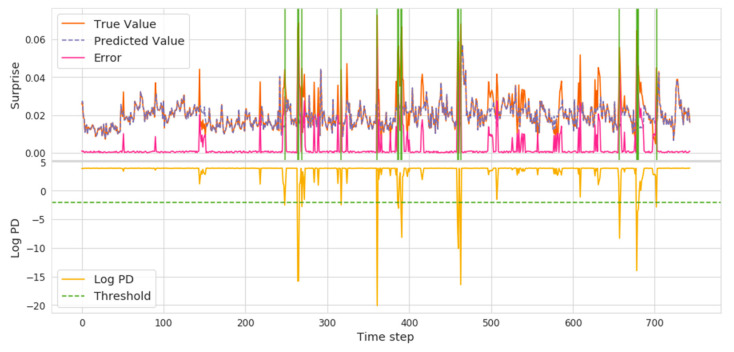
Surprise Volatility and Abrupt Time Points.

Based on the results of our previous analysis, hate, anxiety, and surprise were found to be attractive because they are different from other emotions. In our model detection results (shown in Figure 7, Figure 8 and Figure 9), the lower yellow part represents the threshold position based on the logPD, the orange and purple mark shows the real and predicted emotional valence, and the error is expressed as a shaded green area where there is the abnormal time interval.

Hate has an obvious valence rise process, and the overall trend of volatility shows several key event detections in Figure 7. In the position of the *x* axis around 380, the first higher position of hate, the National Health Commission did not rule out the possibility of human-to-human transmission and thus triggered a significant hate valence increase among the public. Then, around the 490 position, the emotion surge was reignited owing to the human-to-human transmission. The follow-up was identified around position 580, when the global high-risk events were defined by the WHO, and the Red Cross Society reported more events. The positions of the emotional time points were seen to correspond completely with the pandemic public opinions.

As pointed out in Section 2, anxiety was the main emotion seen in public psychology during COVID-19. We can see in Figure 8 that the overall trend is gradually decreasing and slowing down. The initial spike occurred on the timeline around 160, when the WHO declared it was not SARS and MERS. Subsequently, news of “human-to-human” and news of bat origin sparked a rise that led to public anxiety.

Although the intuitive sense in COVID-19 is the dominance of negative emotions, our government is always issuing policies, opening information, and vigorously promoting positive emotions. Surprise, the volatility varying greatly in Figure 9, shows the public exhibited surprise at the timely release of prevention and control measures. 

Many spikes and abnormalities appear after each data update and the diagnosis treatment, which indicates that the public paid attention to the world around them as well as the pandemic prevention plan when quarantined at home. In addition, we found that the valence at night (in a day) is much greater than during daytime trading. Although the government announced relevant news and policies in the daytime, the media generally reported it to the public on the evening news. Considering the spread of information transmission, the fluctuations of public emotions were especially apparent between 10:00 p.m. and 12:00 p.m. This situation was identified in all eight emotions.

### 4.2. Tracking Effect of Triggered Causes

**Figure 10:**
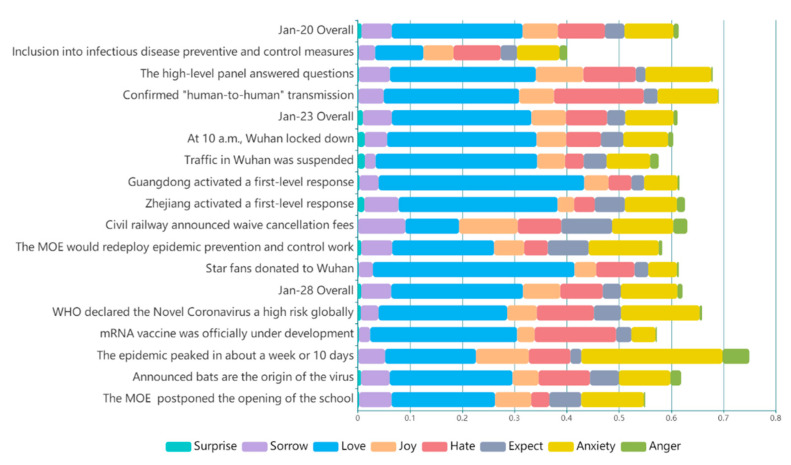
Emotional Distribution of the Days and Public Events.

We show that the emotional distribution of events is an important factor that can be traced back to the overall emotional response on each particular day. We can also visualize the distribution of eight-dimensional emotions (as shown in Figure 10). In particular, on 23 January, the daily distribution was almost the same as that of the Wuhan closure event, with a KL value of 0.0098, which indicates that this event was the biggest factor affecting the public emotion on that day. 

Table 4 illustrates the ranking of typical public opinion events corresponding to emotional arousal paths. We randomly selected three days of candidate events to demonstrate the validity of the emotional path.

From the perspective of tracking triggered events, emotional valence can only be calculated from the content, and the similar emotional distribution can be accurately tracked to locate induced events. However, the premise exists that we have adequate candidate events; otherwise, there would be a lack of content of rank and the track of reason. 

Therefore, we solved the problem of insufficient data in the form of an emotion arousal path by monitoring the abnormal emotions. Based on the comparison of candidate events and target topics from the emotion arousal path over the preceding three days, we compared the results after human cognition of emotional psychology (Table 5). This showed the ranking to be effective and similar to the actual situation using this cognitive method.

### 4.3. Correlation between the Number of Infected People and Emotions Exhibited

To more accurately measure the role of specific discrete emotions in the study, we explored the emotional volatility through algorithms and found the spike point in a previous study. Next, we attempted to find the exact discrete emotions according to the number of infected people. For this, we used data on the number of infected persons released by the National Health Commission (since 13 January). We compared the Pearson correlation coefficient between infected persons and discrete emotions and found some interesting phenomena.

**Figure 11:**
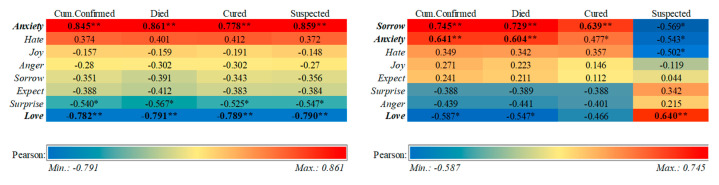
Heat map of correlation between the number of infected people and emotions exhibited. ** *p* < 0.01, * *p* < 0.05.

We first construct the correlation heat map for January in Figure 11 (shown on the left). The deeper shade of red represents positive correlation, and the deeper shade of blue represents negative correlation. By comparing the cumulative number of infected persons, the number of deaths, the number of cured persons, and the number of suspected cases, the calculation results showed that anxiety was significantly positively correlated with the number of infected persons, love was significantly negatively correlated (*p* < 0.01), and surprise was negatively correlated with the number of infected persons (*p* < 0.05). When time shifts to late January, on 31 January, the total number of confirmed cases was over 10,000, while the number of suspected cases exceeded 20,000. 

Next, we show the correlation for February in Figure 11 (shown on the right), and the calculation results show that sorrow and anxiety were positively correlated with the cumulative number of infections, while love was still significantly negatively correlated. However, there was an inversion of the results in suspected cases, and love presented a significant positive correlation. This conclusion suggests that in the initial stage of the pandemic, anxiety was dominant among the public because of the sudden incidence and strange variations caused. However, as the pandemic continued for a period of time, the increase in the number of confirmed cases led to increased sadness, and the correlation of anxiety decreased from the perspective of numerical and color responses. At the same time, the increase in suspected cases also indicated that the suspected cases were still under medical observation and not confirmed, which meant that the country was stepping up nucleic acid testing, in addition to implementing prevention and control efforts, and so the psychological feeling of security promoted correlation of the positive emotions.

## 5. Discussion

Our research summarized the evolution the law of public emotions in public health emergencies. However, the labeled anomaly of the emotion abrupt time point is rare, which is always a major challenge to the anomaly detection task. Moving forward, we will stick to our social-emotional perception model to discover other potentials in public health, and conduct extensive experiments on other domains and tasks, such as public safety, to further validate the model’s feasibility and stability.

## 6. Conclusions

Our study focused on monitoring and measuring public emotions embedded in the social media context during public health emergencies, and provided three contributions: (1) the construction of a discrete emotion lexicon representing the public under the state of the COVID-19 pandemic, (2) the accurate detection of emotion abrupt time points for different discrete emotions affecting the public, and (3) the location of specific emotional causes that induced abnormal emotion following the emotional arousal path, using the OCC emotion model. The public emotional perception model has a more prepared traceability scheme for the induced causes and has confirmed that information disclosure including prevention and control measures have driven positive emotions, such as love and expect. 

## Figures and Tables

**Table 1:** Coverage Comparison in Different Lexicons and Valences.

	Original	Expansion	≥0.1	≥0.2	≥0.3	≥0.4	≥0.5	≥0.6	≥0.7	≥0.8	≥0.9
**Count in Lexicon**	17,530	45,096	29,681	24,044	20,601	16,892	13,950	9052	4541	2002	458
**Coverage in Corpus**	0.1485	0.7484	0.6531	0.5186	0.4825	0.4129	0.3371	0.2431	0.1645	0.1292	0.0835
**Count in Domain**	24	1837	1625	1164	1044	833	600	432	186	75	19
**Coverage in Domain**	0.0097	0.7482	0.6619	0.4741	0.4252	0.3393	0.2443	0.1759	0.0757	0.0305	0.0077

**Table 2:** Seven Topics and Three Emotional Arousal Paths.

Topics	Emotional Arousal Path
Pandemic news and data report	Aspects of Objects
Medical care was on the front line	Actions of Agents
Nation- and local-issued policy measures	Consequence of Events
Public protection initiative	Aspects of Objects
Scientific breakthroughs and knowledge dissemination	Consequence of Events
Peripheral symptoms cause inner anxiety	Actions of Agents
Be objective in daily life	Aspects of Objects

**Table 3:** Performance Comparison of Different Thresholds.

log PD	Predictive Abrupt Points	True Value		Accuracy	Recall
		Abnormal	Normal		
−1.0	24	13	11	0.541	0.812
−1.5	17	12	5	0.705	0.75
−2.0	13	10	3	0.769	0.625
−2.5	9	7	2	0.777	0.437
−3.0	4	4	0	1.0	0.25

**Table 4:** Performance of KL Value of Candidate Events for Three Days.

Time Point	Emotion Type	Public Opinion Events	Topics	Path	KL Value
**20-Jan**	surprise sorrow joy hate anxiety	Inclusion into class B infectious disease and class A preventive and control measures	Nation and local issued policy measures	Consequence of Events	0.0982
		The high-level panel of the National Health Commission answered questions	Epidemic news and data report	Aspects of Objects	0.0375
		National Health Commission confirmed “human-to-human” transmission	Scientific breakthroughs and dissemination	Consequence of Events	0.0674
**23-Jan**	anger joy expect hate	At 10 a.m., Wuhan locked down	Nation and local issued policy measures	Consequence of Events	0.0098
		Traffic in Wuhan was suspended	Nation and local issued policy measures	Consequence of Events	0.0786
		Guangdong activated a first-level response to a major public health emergency	The public took the initiative to protection	Aspects of Objects	0.0907
		Zhejiang activated a first-level response to a major public health emergency	The public took the initiative to protection	Aspects of Objects	0.0667
		Civil railway announced waive cancellation fees	State and local issued policy measures	Consequence of Events	0.2835
		The Ministry of Education would redeploy epidemic prevention and control work	State and local issued policy measures	Consequence of Events	0.0747
		Star fans donated to Wuhan	Medical care was on the front line	Actions of Agents	0.0992
**28-Jan**	surprise hate expect anxiety	WHO declared the Novel Coronavirus a high risk globally	Epidemic news and data report	Aspects of Objects	0.0354
		Novel coronavirus mRNA vaccine was officially under development	State and local issued policy measures	Consequence of Events	0.1568
		The epidemic peaked in about a week or 10 days	Scientific breakthroughs and dissemination	Consequence of Events	0.1736
		The Chinese Academy of Medical Sciences announced that bats are the origin of the virus	Scientific breakthroughs and dissemination	Consequence of Events	0.0245
		The Ministry of Education postponed the opening of the school	State and local issued policy measures	Consequence of Events	0.0651

**Table 5:** The Ranking Comparison of Events and Emotional Paths.

Abrupt Time Point	Events in Media	Events in Dataset	Topics	Event MAP	Topic MAP
20-Jan	6	3	3	0.333	0.667
23-Jan	9	7	3	0.429	1
28-Jan	18	5	3	0.6	0.667

## Data Availability

The data can be found here: ai.suibe.net (accessed on 22 April 2021).
